# DUSP10 is a novel immune-related biomarker connected with survival and cellular proliferation in lower-grade glioma

**DOI:** 10.18632/aging.204821

**Published:** 2023-06-29

**Authors:** Feng Xiao, Hong Zhu, Yun Guo, Zhe Zhang, Gufeng Sun, Kai Huang, Hua Guo, Guowen Hu

**Affiliations:** 1 Department of Neurosurgery, The Second Affiliated Hospital of Nanchang University, Nanchang 330006, Jiangxi, China; 2 Jiangxi Key Laboratory of Neurological Tumors and Cerebrovascular Diseases, Nanchang 330006, Jiangxi, China; 3 Jiangxi Health Commission Key Laboratory of Neurological Medicine, Nanchang 330006, Jiangxi, China; 4 Institute of Neuroscience, Nanchang University, Nanchang 330006, Jiangxi, China

**Keywords:** DUSP10, lower-grade glioma, prognosis, immunotherapy, chemotherapy, cell proliferation

## Abstract

Objective: The role of dual-specificity phosphatase 10 (DUSP10) has been investigated in several types of cancer. Nevertheless, the underlying function of DUSP10 in lower-grade glioma (LGG) remains undetermined.

Methods: We entirely determined the expression features and prognostic significance of DUSP10 in numerous tumors by implementing a pan-cancer analysis. Adjacently, we thoroughly inspected the correlation between DUSP10 expression and clinicopathologic features, prognosis, biological processes, immune traits, gene variations, and treatment responses based on the expression features in LGG. *In vitro* studies were conducted to detect the underlying functions of DUSP10 in LGG.

Results: Unconventionally boosted DUSP10 expression and higher DUSP10 expression correlated with poorer prognosis were discovered in various tumors, including LGG. Fortunately, DUSP10 expression was proven to be an independent prognostic indicator of patients with LGG. Additionally, DUSP10 expression was tightly linked to the immune modulation, gene mutations, and response to immunotherapy/chemotherapy in LGG patients. *In vitro* studies illustrated that the DUSP10 was abnormally increased and pivotal for cell proliferation in LGG.

Conclusions: Collectively, we verified that DUSP10 was an independent prognostic indicator and may become a novelty target of targeted therapy of LGG.

## INTRODUCTION

Gliomas are the most common brain tumors [1]. In line with the corresponding World Health Organization (WHO)-standard, gliomas are classified from grade I to grade IV [2]. Importantly, grade II and III gliomas are considered LGGs by the Cancer Genome Atlas (TCGA). LGGs could be classified on the basis of the clinicopathologic features, such as the 1p/19q codeletion and isocitrate dehydrogenase (IDH) mutation status [3]. Although LGGs patients possess better prognosis, 70% of them will develop malignant progression within 10 years [4]. Currently, the most common clinical management strategies, including surgery, chemotherapy, and radiotherapy, are applied to treat LGG [5]. Nevertheless, the number of clinical anticancer drugs used to treat patients with LGG has remained scarce, and the clinical prognosis of LGG patients is still not satisfactory. Thence, it is imminently needed to examine novel molecular signatures for the evaluation of the prognosis and individualized treatment of patients with LGG.

DUSP10, also named MKP5, plays a vital part in cell growth and proliferation [6]. DUSP10 protein exists as two Cdc25 homology regions: a specific 150 N-terminal amino acid sequence and a C-terminal catalytic domain [7]. Recent research has revealed that elevated DUSP10 expression was strictly associated with the malignant development of several cancers, such as liver [8], colorectal [9], breast [10], and pancreatic [11] cancers. Nevertheless, the specific role of DUSP10 in patients with LGG is still unknown. Thus, we implemented bioinformatic analysis and *in vitro* studies verification to inspect the underlying features of DUSP10 in LGG patients. This research may be significant for evaluating the survival and excavating effective therapies for LGG patients.

Firstly, we initiated a pan-cancer analysis of DUSP10 and determined that the prognostic significance of DUSP10 in pan-LGG was more valuable than in other tumors. Thus, it was necessary to implement further study to examine the prognostic significance of DUSP10 in LGG. Subsequently, we utilized the three independent LGG cohorts, including the TCGA, Chinese Glioma Genome Atlas (CGGA), and GSE61374 cohorts, to further inspect the prognostic significance of DUSP10 in LGG. In line with the median values of DUSP10 expression, we categorized the LGG patients into high- and low-DUSP10 expression subtypes. Survival analysis verified that high-DUSP10 subset possessed worse prognosis than low-DUSP10 subset. Multiple studies have illustrated that tumor immune microenvironment may be strongly related to the survival of patients with tumors [12, 13]. Therefore, we estimated the connection between DUSP10 expression and immunological traits (including stromal and immune scores, tumor-infiltrating immune cells [TIICs], and immune checkpoint genes [ICPGs] expression), genomic alternations, and responses to immunotherapy/chemotherapy. Afterwards, we conducted the *in vitro* studies to ascertain the anomalous expression and the underlying functions of DUSP10 in LGG. In short, we considered that DUSP10 was an independent prognostic indicator and may play a critical part in the treatment of LGG patients based on the above-presented comprehensive analysis.

## RESULTS

### Pan-cancer analysis of DUSP10

**Figure 1:**
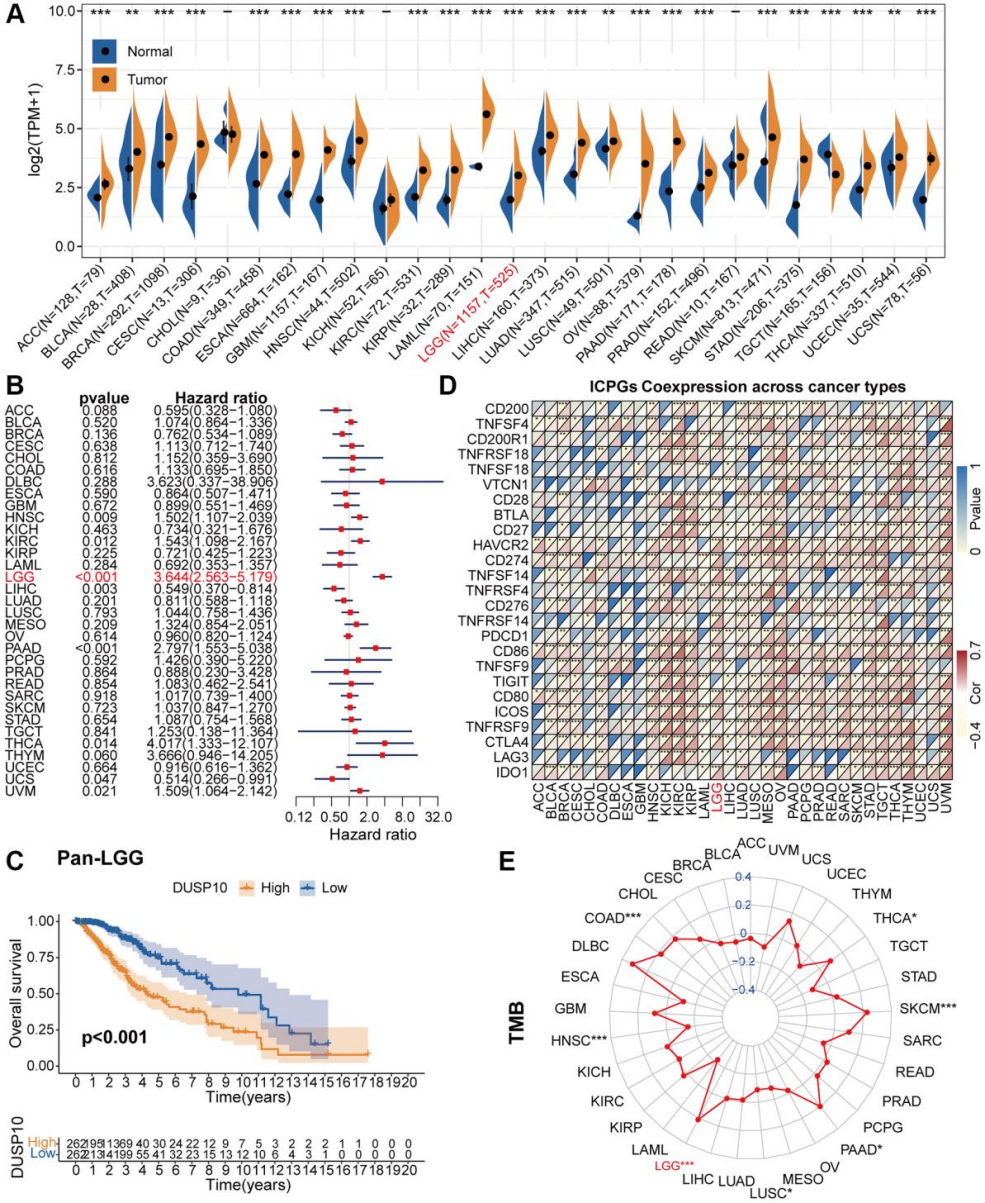
**Pan-cancer analysis of DUSP10.** (**A**) Differential expression of DUSP10 in normal and cancer tissues. (**B**) Univariate Cox regression analysis of DUSP10 expression in various tumors. (**C**) Kaplan-Meier analysis of DUSP10 in pan-LGG. (**D**) Co-expression of DUSP10 and ICPGs in different cancers. (**E**) Differential TMB in diverse cancers. ^*^P < 0.05, ^**^P < 0.01, ^***^P < 0.001.

The flow diagram of the research was exhibited in Supplementary Figure 1. By contrasting the DUSP10 expression data of pan-cancer, we discovered that DUSP10 was aberrantly increased in diverse tumors, including LGG (Figure 1A). Forest charts exhibited that increased DUSP10 expression was inversely connected with overall survival (OS) in HNSC, KIRC, LGG, LIHC, PAAD, THCA, UCS, and UVM (Figure 1B). Additionally, the results of survival analysis also demonstrated that higher DUSP10 expression tended to demonstrate a worse prognosis in pan-LGG (Figure 1C).

Afterwards, we excavated the interrelation between DUSP10 expression and ICPGs expression in 33 tumors. The co-expression results testified that DUSP10 was tightly correlated with most of ICPGs in pan-LGG (Figure 1D). Moreover, we inspected the connection between DUSP10 expression and tumor mutation burden (TMB) in 33 types of tumors. In COAD, LGG, PAAD, SKCM, and THCA, DUSP10 expression was positively linked to TMB, whereas in HNSC and LUSC, it was inversely linked to TMB (Figure 1E).

### Correlation between DUSP10 and clinicopathologic features in LGG

**Figure 2:**
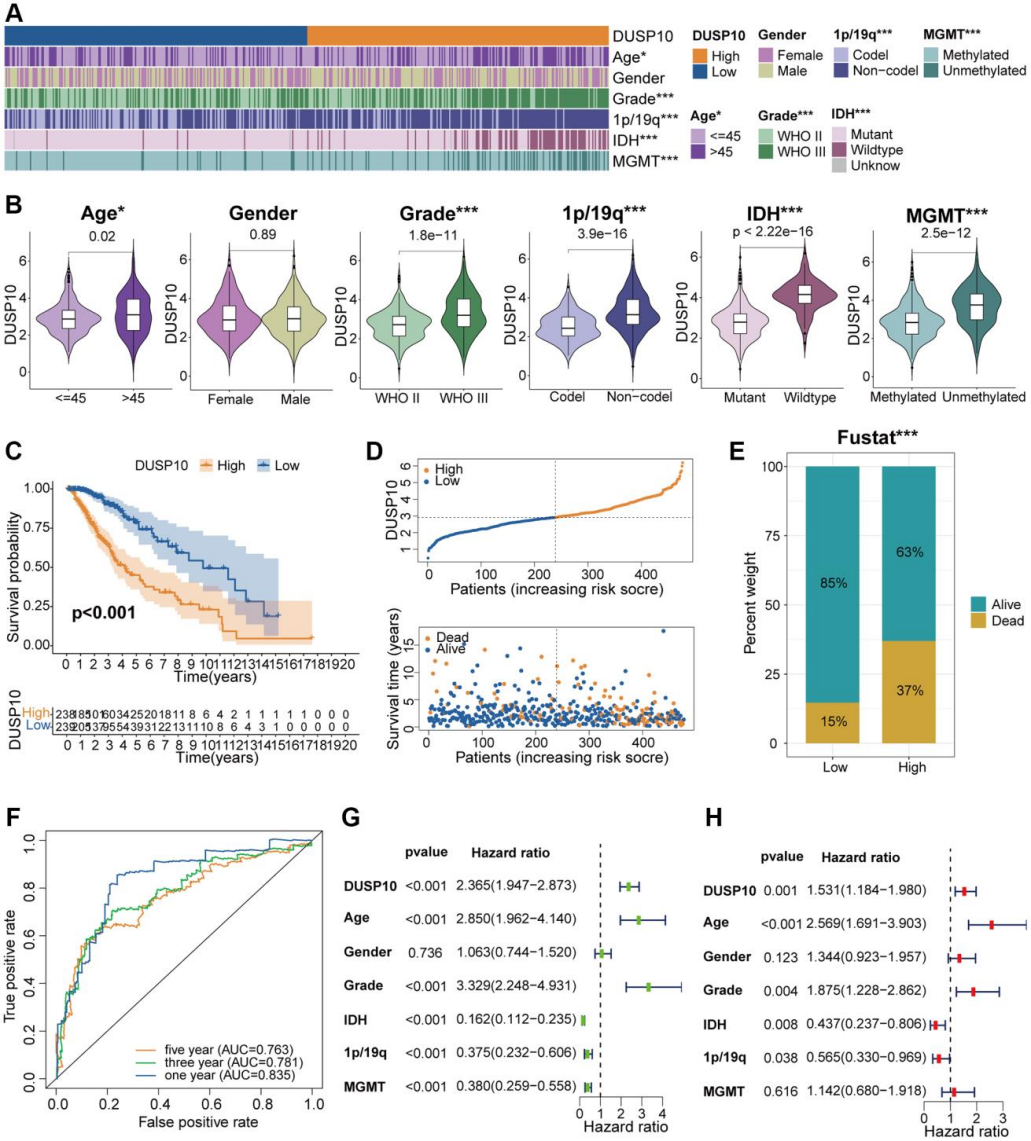
**Clinical correlation analysis of DUSP10 in TCGA.** (**A**) Association between DUSP10 expression and clinical traits of LGG in TCGA. (**B**) Variance analysis of DUSP10 expression in different clinical features (including age, gender, grade, and 1p/19q, *IDH*, and MGMT statuses) in the TCGA dataset. (**C**) Prognostic analysis of high-DUSP10 and low-DUSP10 subtypes in the TCGA dataset. (**D**) Distribution of risk score, OS, and OS status of high-DUSP10 and low-DUSP10 subtypes in the TCGA dataset. (**E**) Different proportions of the living situation between the two subtypes. (**F**) ROC curves representing the predictive role of the risk score in TCGA. (**G**, **H**) Univariate and multivariate Cox analyses of DUSP10 expression and clinicopathological characteristics in TCGA. ^*^P < 0.05, ^**^P < 0.01, ^***^P < 0.001.

In line with the median values of DUSP10 expression, we isolated LGG samples into high-DUSP10 and low-DUSP10 subsets and inspected the interrelation between DUSP10 expression and clinicopathologic properties in the three datasets. The results declared that up-regulated DUSP10 expression was obviously linked to older age, 1p/19q non-codel, *IDH* wildtype, and MGMT unmethylation in the TCGA dataset (Figure 2A, 2B). Analogical outcomes were examined in CGGA (Supplementary Figure 2A, 2B) and GSE61374 (Supplementary Figure 3A, 3B) datasets.

### Elevated DUSP10 expression correlates with poor prognosis of LGG

The Kaplan-Meier (KM) analysis was applied to explore the differential OS prognosis between the two subtypes in LGG patients. The results illustrated that the OS of high-DUSP10 subset was apparently worse than low-DUSP10 subset in the TCGA (Figure 2C), CGGA (Supplementary Figure 2C), and GSE61374 (Supplementary Figure 3C) datasets. Therefore, we studied the association between DUSP10 expression, risk score, and OS status in LGG patients and discovered that up-regulated DUSP10 expression was related to the higher risk score and worse OS status in TCGA (Figure 2D), CGGA (Supplementary Figure 2D), and GSE61374 (Supplementary Figure 3D) datasets. The detailed proportion of survival status of LGG samples was also examined in the TCGA (Figure 2E), CGGA (Supplementary Figure 2E), and GSE61374 (Supplementary Figure 3E) datasets. Additionally, the area under the curves (AUCs) for 1/3/5-year OS were 0.835, 0.781, and 0.763, respectively, in the TCGA dataset (Figure 2F); 0.755, 0.796, and 0.755, respectively, in the CGGA dataset (Supplementary Figure 2F); and 0.647, 0.736, and 0.648, respectively, in GSE61374 dataset (Supplementary Figure 3F).

### Independent prognostic significance of DUSP10 in LGG

Univariate and multivariate Cox regression analyses were executed to evaluate whether DUSP10 was an independent prognostic factor in the three datasets. The results indicated that DUSP10 expression, WHO grade, *IDH*, age, and 1p/19q were independent prognostic indicators of LGG patients in the TCGA cohort (Figure 2G, 2H). In the CGGA dataset, we detected that DUSP10 expression, WHO grade, and 1p/19q were independent prognostic indicators of LGG patients (Supplementary Figure 2G, 2H). Similarly, in the GSE61374 cohort, DUSP10 expression, age, and 1p/19q were also considered independent prognostic indicators of LGG patients (Supplementary Figure 3G, 3H). Thence, DUSP10 expression may be an independent prognostic indicator of LGG patients.

### Functional annotations of DUSP10

**Figure 3:**
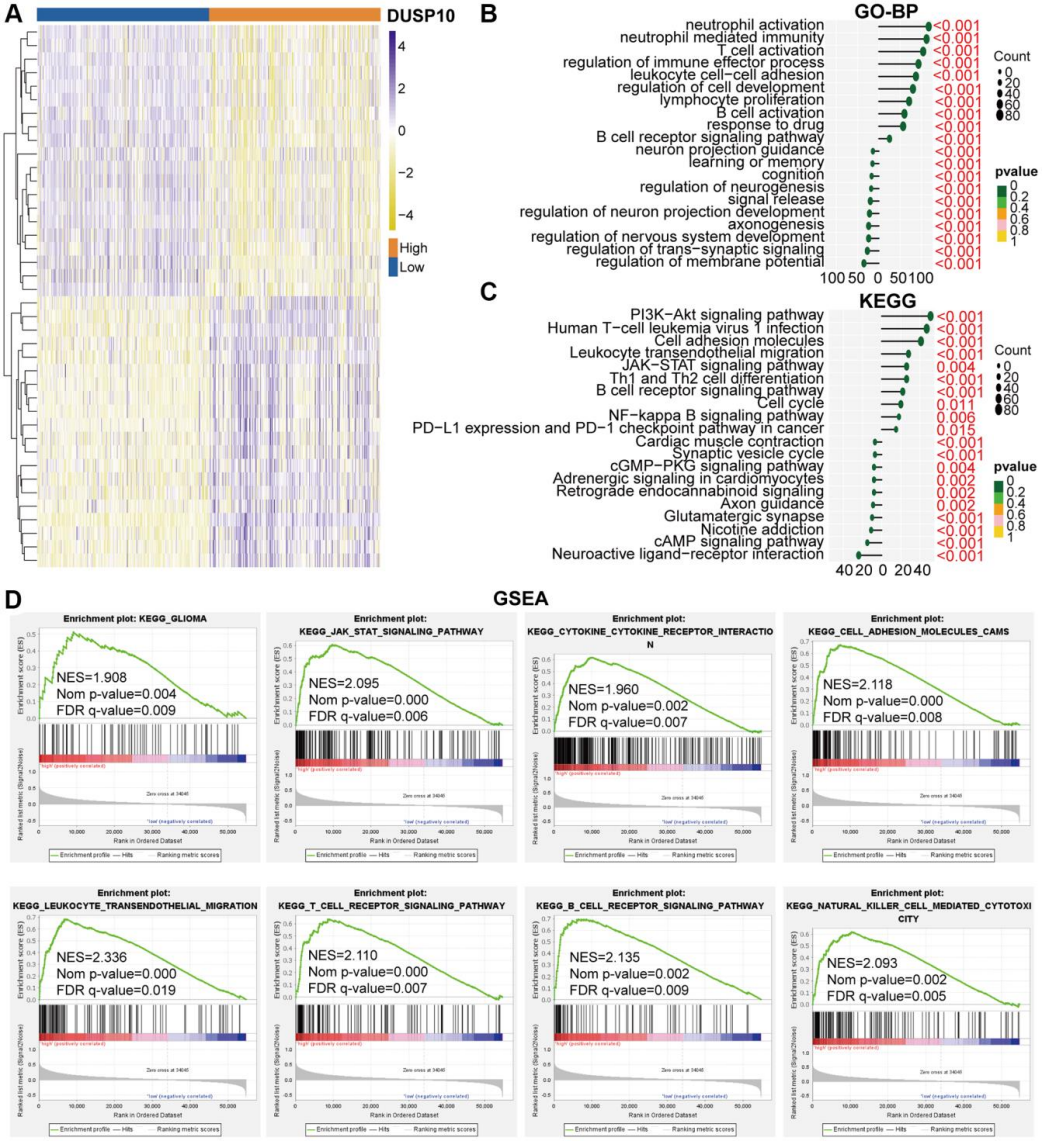
**Biological functions of DUSP10 in LGG in TCGA.** (**A**) DEGs between the low-DUSP10 and high-DUSP10 expression LGG subgroups. (**B**, **C**) The GO-BP (**B**) and KEGG (**C**) analyses for DUSP10 in LGG patients in the TCGA dataset. (**D**) GSEA in the TCGA dataset.

We ascertained differentially expressed genes (DEGs) on the basis of the mean values of DUSP10 expression (|log2 (fold change)| >0.5 and *P* < 0.05). In total, we screened out 461 down-regulated and 1819 up-regulated DEGs in the TCGA cohort and 637 down-regulated and 1954 up-regulated DEGs in the CGGA cohort. Conspicuous DEGs in TCGA (Figure 3A) and CGGA (Supplementary Figure 4A) cohorts were displayed in the heatmap. Subsequently, these down-regulated and up-regulated DEGs were exploited to conduct Gene Ontology biological process (GO-BP) and Kyoto Encyclopedia of Genes and Genomes (KEGG) analyses. In the TCGA dataset, the GO-BP results of down-regulated DEGs indicated that down-regulated expression of DUSP10 was apparently associated with the regulation of nervous system development, regulation of trans-synaptic signaling, learning or memory, regulation of neurogenesis, and cognition. Additionally, up-regulated genes were majorly enriched in neutrophil activation, T cell activation, and response to the drug (Figure 3B). Analogical results were ascertained in the CGGA dataset (Supplementary Figure 4B). The KEGG analysis in the TCGA (Figure 3C) and CGGA (Supplementary Figure 4C) datasets showed that down-regulated DEGs were enriched in neuroactive ligand-receptor interaction, and synaptic vesicle cycle, and up-regulated DEGs were mostly enriched in the PI3K-Akt and JAK-STAT signaling pathways, leukocyte transendothelial migration, and cell cycle.

GSEA analysis was implemented to examine the underlying molecular mechanisms between high-DUSP10 and low-DUSP10 subtypes in LGG. The results prompted that the high-DUSP10 subtype was majorly linked to the JAK-STAT signaling pathway, and leukocyte transendothelial migration in the TCGA dataset (Figure 3D). Analogical outcomes were found in the CGGA cohort (Supplementary Figure 4D).

### Connection between DUSP10 and immune traits

**Figure 4:**
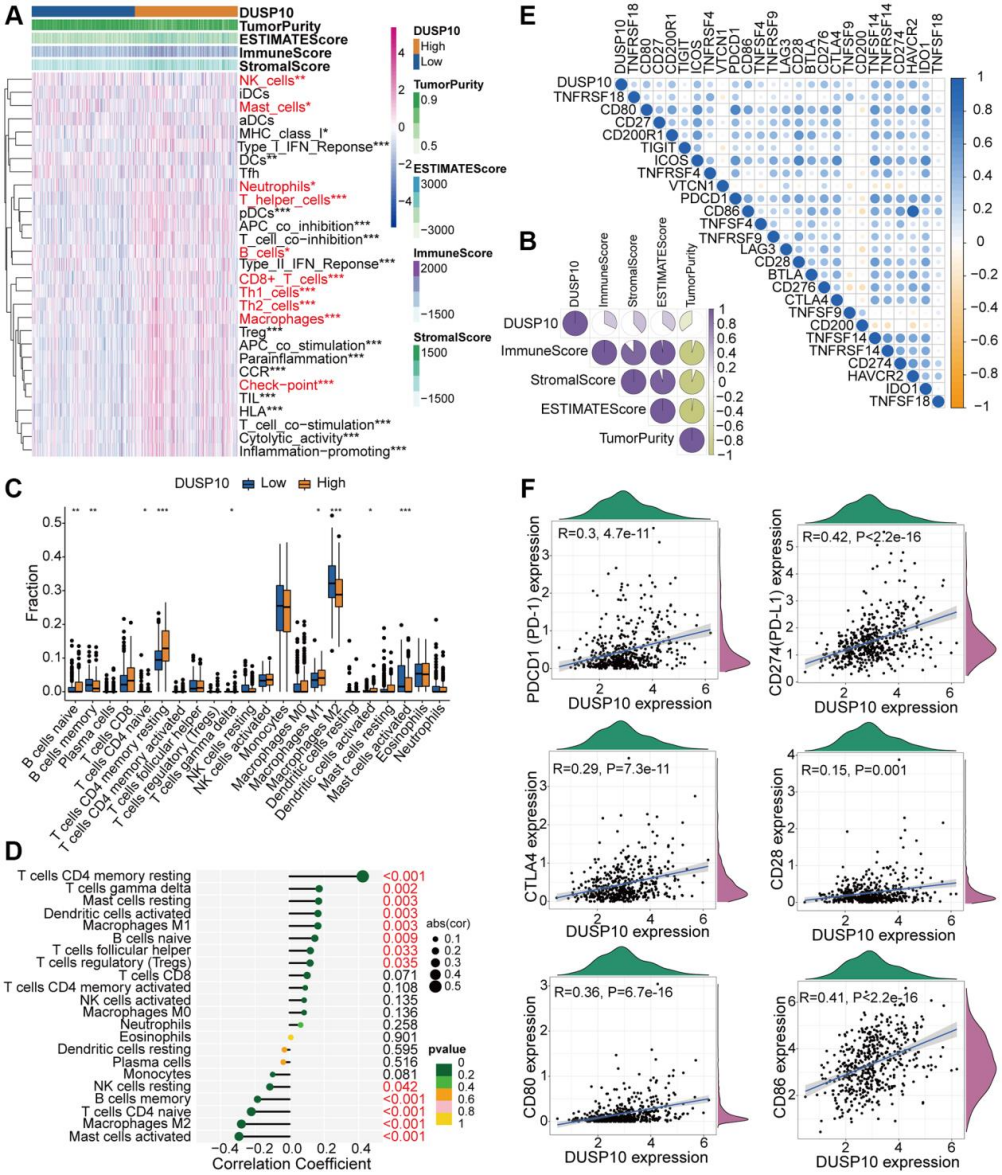
**Different TME and immunological characteristics of the low-DUSP10 and high-DUSP10 subtypes in TCGA.** (**A**, **B**) Association between DUSP10 expression and 29 immune-associated signatures, ESTIMATE, immune, stromal scores, and tumor purity. (**C**) Comparisons of infiltration of 22 types of immune cells in the two subgroups. (**D**) Lollipop plots exhibited the relationship between DUSP10 expression and TIICs. (**E**, **F**) Co-expression analysis of DUSP10 and 25 ICPGs. ^*^P < 0.05, ^**^P < 0.01, ^***^P < 0.001.

The results of functional enrichment analysis exhibited the underlying association between DUSP10 and immune regulation in LGG. This urged us to estimate the interrelation between DUSP10 and immune traits in LGG. We adopted the ssGSEA algorithm to recognize the abundance of 29 immune-connected indicators to check the interrelation between DUSP10 expression and immune infiltration. The immune-associated signatures in low-DUSP10 subset were apparently lower than in high-DUSP10 subset in the TCGA (Figure 4A) and CGGA (Supplementary Figure 5A) datasets. The results disclosed that DUSP10 expression was positively related to the ESTIMATE, stromal and immune scores but inversely linked to tumor purity in the TCGA (Figure 4B) and CGGA (Supplementary Figure 5B) datasets. Moreover, we conducted the CIBERSORT algorithm to investigate the infiltration abundances of TIICs between the two subtypes. The infiltration abundances of resting memory CD4^+^ T cells, macrophages M1, and naive B cells were positively linked to DUSP10 expression, and macrophages M2, naive CD4^+^ T cells, and memory B cells were inversely related to DUSP10 expression in the TCGA cohort (Figure 4C, 4D). We discovered similar results from the CGGA dataset (Supplementary Figure 5C, 5D).

Additionally, we also confirmed that DUSP10 expression was positively connected with the majority of ICPGs in the TCGA dataset (Figure 4E). The detailed correlation between DUSP10 and some celebrated ICPGs (including PD1, PD-L1, CTLA4, CD28, CD80, and CD86) was explored by conducting a correlation analysis in the TCGA cohort (Figure 4F). The above-mentioned outcomes were also determined in the CGGA dataset (Supplementary Figure 5E, 5F). Moreover, we examined the expression distribution of DUSP10 among distinct cell types in LGG immune microenvironment by exploiting GSE167960 dataset and found that DUSP10 was most elevated in immune cells (Supplementary Figure 6).

### DUSP10 expression is associated with gene alterations

**Figure 5:**
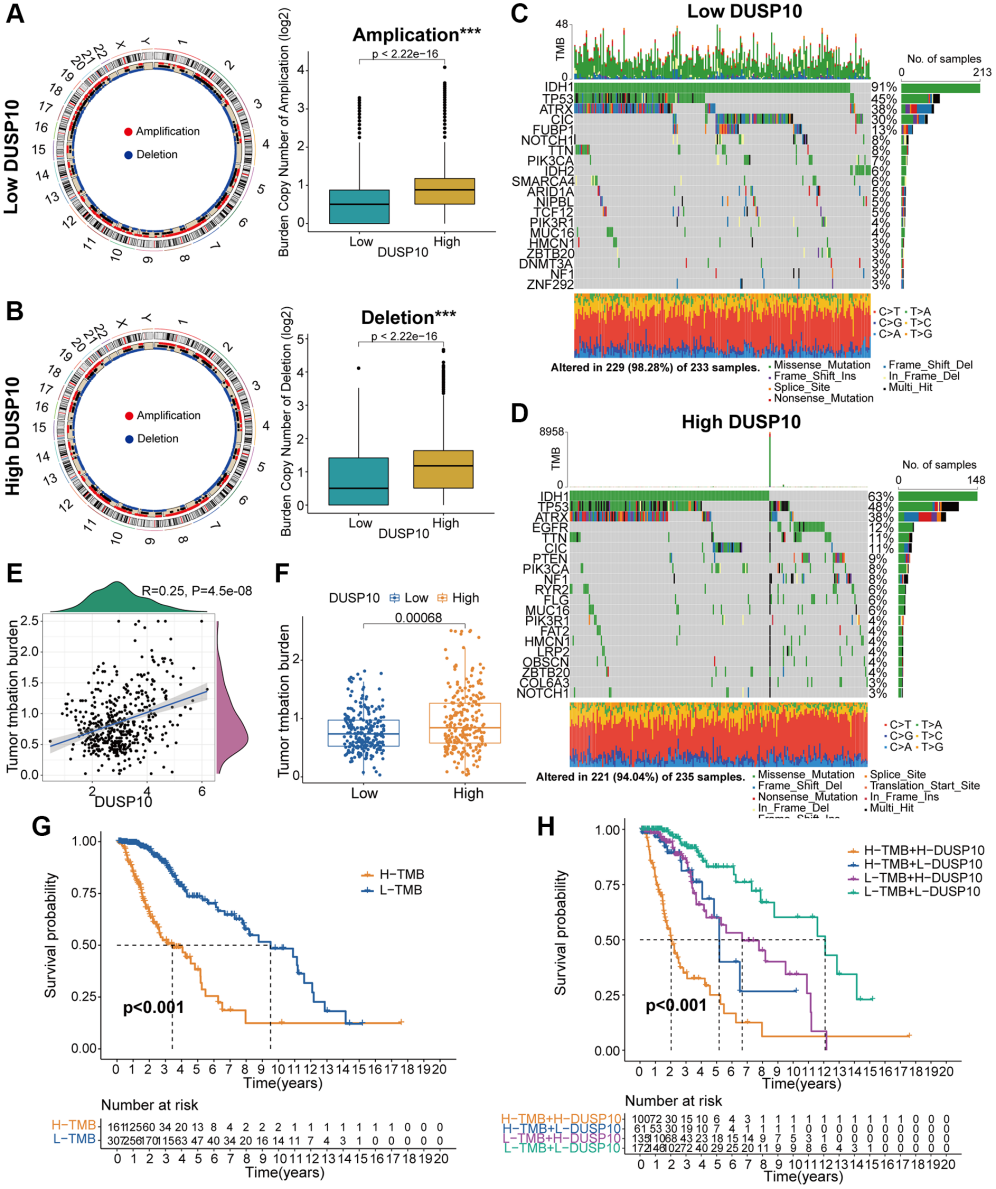
**Contradistinction of genomic mutations between the two subgroups in the TCGA dataset.** (**A**, **B**) Circos plots of low- and high-DUSP10 subgroups illustrated the amplifications and deletions of chromosomes, and boxplots exhibited a lower burden of copy number amplifications and deletions in the low-DUSP10 subgroup. (**C**, **D**) Waterfall plots revealed mutated genes in the low-DUSP10 (**C**) and high-DUSP10 (**D**) subgroups. (**E**, **F**) An association between DUSP10 expression and TMB levels. (**G**, **H**) Relationship between TMB level and the prognosis of patients with LGG (**G**) and the differential prognostic value in the two subtypes with distinct TMB level (**H**). ^*^P < 0.05, ^**^P < 0.01, ^***^P < 0.001.

An accumulating body of research has disclosed that genomic variations might play a vital part in adjusting immune infiltration and tumor immunity [14, 15]. Thus, we employed copy number alteration (CNA) and somatic mutations analysis to recognize gene variations between low-DUSP10 and high-DUSP10 subsets. The frequency of CNA, both amplification and deletion, was obviously lower in low-DUSP10 subset than in high-DUSP10 subset (Figure 5A, 5B). Whereafter, the “waterfall” plot was created to exhibit that both low- and high-DUSP10 expression subsets possessed specific mutated genes. The results indicated that the variation frequencies of *IDH1* and *CIC* in low-DUSP10 subset were higher than in high-DUSP10 subset (Figure 5C, 5D). Afterwards, we inspected the interrelation between DUSP10 expression and TMB level in LGG and detected that DUSP10 expression was positively connected with the TMB level (Figure 5E, 5F). The results exhibited that high-TMB subset owned worse OS than low-TMB subset (Figure 5G). Next, we further investigated the differential OS of distinct DUSP10 expression in the low- and high-TMB subsets and discovered that higher DUSP10 expression and TMB level owned worser OS in patients with LGG (Figure 5H).

### Relationship between DUSP10 expression and treatment responses

**Figure 6:**
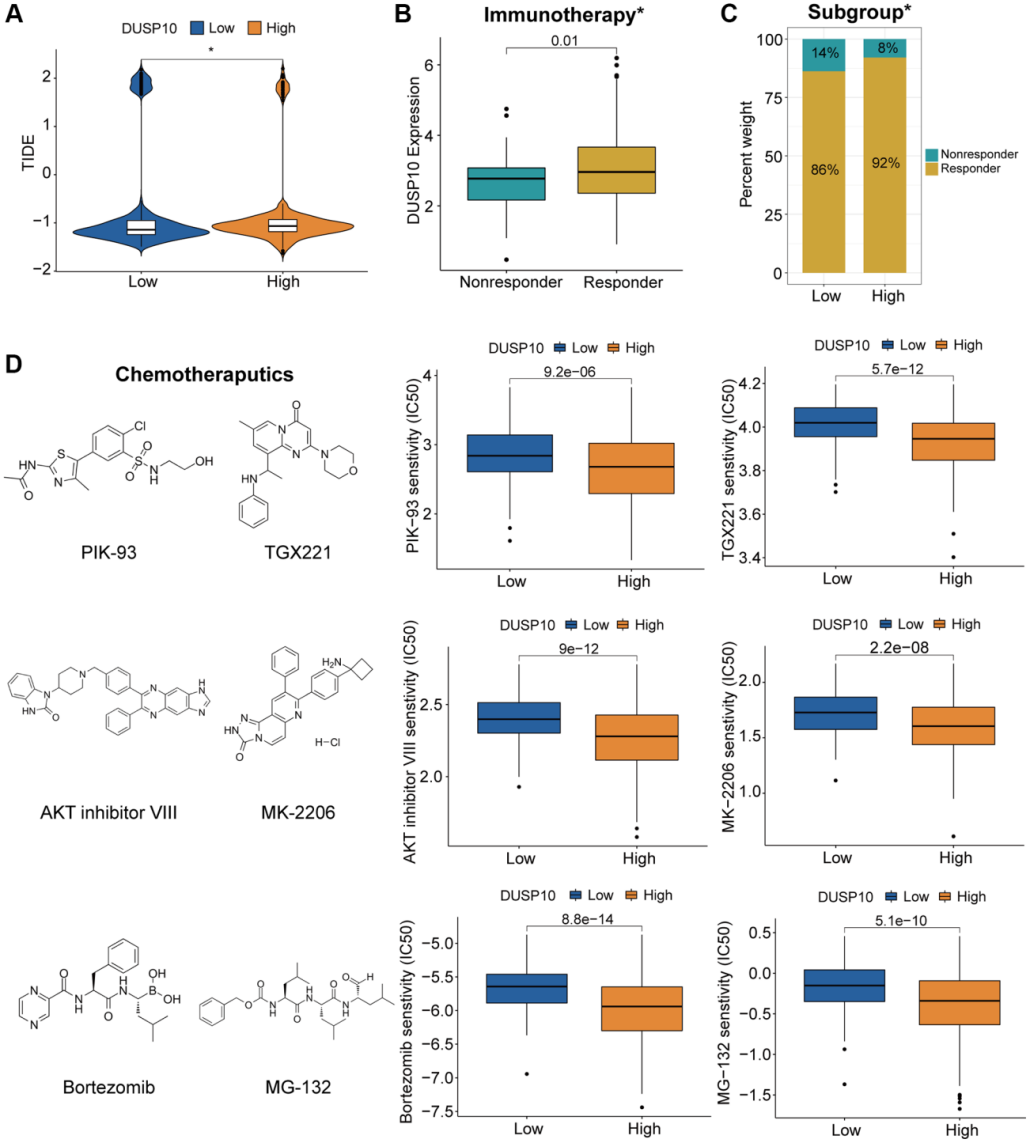
**Different responses to immunotherapy/chemotherapy of the two subgroups in the TCGA dataset.** (**A**) Differential TIDE scores in the two subtypes. (**B**, **C**) Distinct proportions of non-responders and responders to immunotherapy between the two subgroups. (**D**) Prediction of response to chemotherapeutics drugs in different subtypes.

We executed the TIDE algorithm to forecast the reaction of LGG patients to immune checkpoint blockers (ICB) therapy and discovered that the high-DUSP10 subtype acquired better TIDE scores than the low-DUSP10 subtype (Figure 6A). The DUSP10 expression in responders to ICB therapy was higher than in non-responders (Figure 6B). Additionally, the proportion of responders in low-DUSP10 subset was also lower than in high-DUSP10 subset (Figure 6C). Thus, DUSP10 could be a potential immunotherapy target.

We investigated the association between DUSP10 expression and chemotherapeutics (such as PIK-93, TGX221, AKT inhibitor VIII, MK-2206, bortezmib, and MG-132) based on the results of functional annotations to evaluate LGG patients with distinct DUSP10 expression in guiding chemotherapy. The results testified that the high-DUSP10 subgroup was related to lower inhibitory centration (IC50) in these anticarcinogens (Figure 6D). It means that high-DUSP10 subtype was more sensitive to these anticarcinogens.

### *In vitro* experiments of DUSP10 in LGG

**Figure 7:**
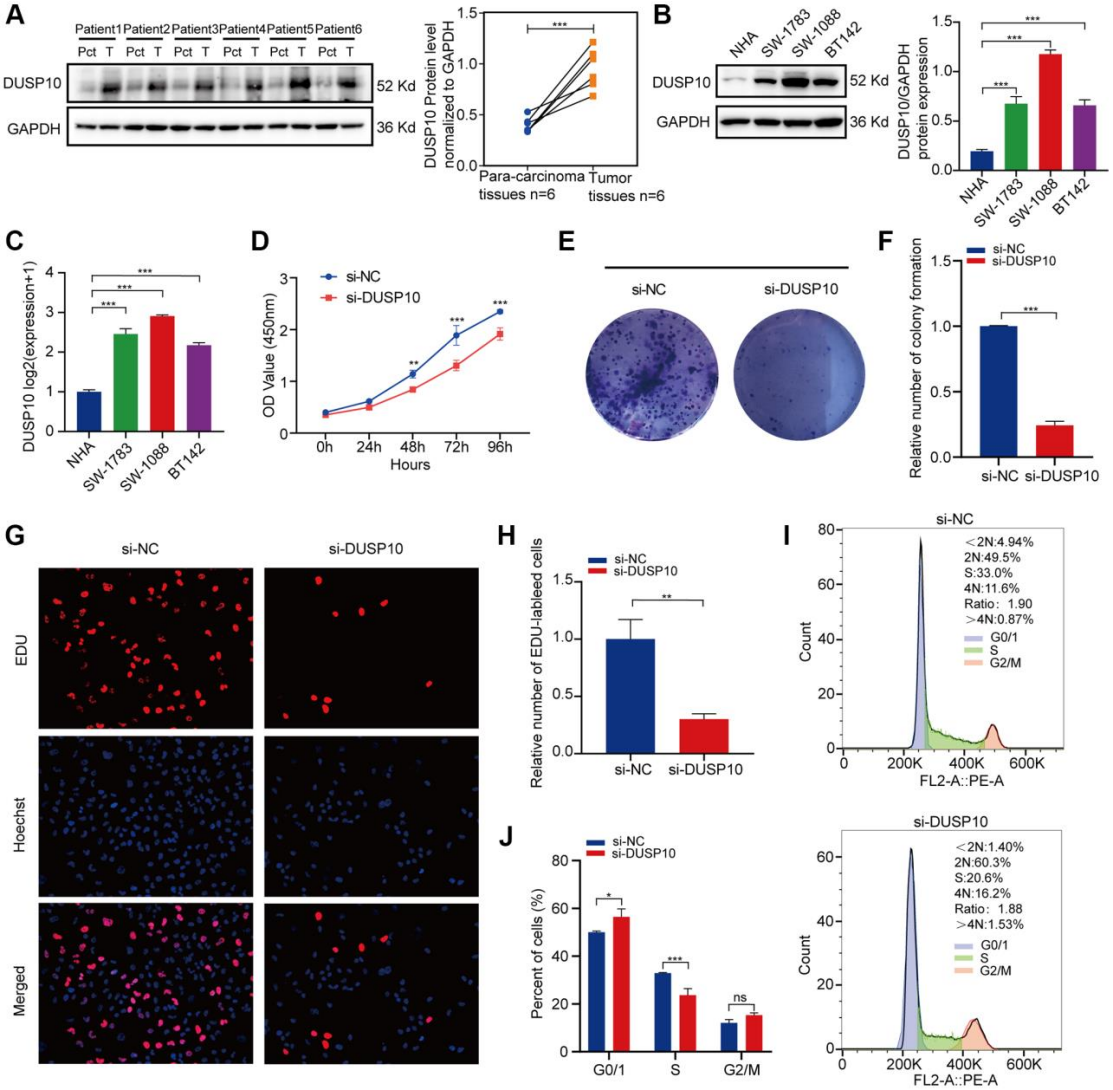
***In vitro* experiments verification of DUSP10 in LGG.** (**A**) Western blot analysis of DUSP10 expression in LGG tissues and corresponding para-carcinoma tissues. (**B**) Western blot and (**C**) qRT-PCR analysis of DUSP10 expression in NHA and LGG cell lines. (**D**) The cell viability of si-DUSP10-transfected and si-NC-transfected SW1088 cells by CCK-8 assays. (**E**, **F**) Effect of DUSP10 knockdown on colony formation was counted in SW1088 cells. (**G**, **H**) Representative images (**G**) and histogram analysis (**H**) of EdU assays after DUSP10 knockdown in SW1088 cells. (**I**, **J**) Cell cycle assays were implemented to evaluate the cell distribution of the SW1088 cell lines transfected with si-DUSP10 or si-NC lentiviruses. ^*^P < 0.05, ^**^P < 0.01, ^***^P < 0.001.

The protein expression levels of DUSP10 were apparently higher in LGG tissues when compared to para-cancerous tissues (Figure 7A). Additionally, we examined the protein and mRNA expression of DUSP10 in three LGG cell lines (including SW-1783, SW-1088, and BT142) and a NHA cell line and discovered that DUSP10 expression was distinctly higher in the LGG cell lines when compared to the NHA cell line (Figure 7B, 7C).

Subsequently, we inspected the interrelation between DUSP10 expression and cell proliferation in LGG. CCK-8 (Figure 7D) and colony formation assays (Figure 7E, 7F) displayed that the proliferative capacity of the SW1088 si-DUSP10 group was markedly reduced when compared to the si-NC group. Meanwhile, downregulating DUSP10 expression resulted in an obvious reduction in proliferation implemented by EdU assays in SW1088 cells (Figure 7G, 7H). In addition, we also observed that the cell cycle was strongly influenced by the downregulation of DUSP10 expression. After knocking down DUSP10 of SW1088 ells, the number of cells in the S phase was reduced and the G0/G1 phase was elevated (Figure 7I, 7J). These results illustrate that DUSP10 play a crucial part in the cell proliferation in LGG.

## DISCUSSION

In spite of great progress has been obtained in LGG surgery, radiotherapy, and chemotherapy, patients with LGG still have poor clinical prognosis [16–18]. Since the effect of traditional treatments for LGG patients remains limited, there is clearly needed to investigate the effective prognostic and therapeutic targets for these patients. DUSP10, a member of the MKPs subfamily, is a crucial factor in regulating cell proliferation. A growing number of studies have elaborated that increased DUSP10 expression may promote the malignant progression of several cancers. Nevertheless, the particular value of DUSP10 in patients with LGG remains unknown. Thus, we entirely investigated the connection between DUSP10 expression, clinical features, prognosis, biological functions, tumor immunity, gene variations, and responses to immunotherapy/chemotherapy in LGG.

We employed the pan-cancer analysis of DUSP10 and detected that higher DUSP10 expression was linked to shorter survival time, higher ICPGs expression, and higher TMB burden in pan-LGG. We implemented survival analysis in the TCGA dataset to estimate the prognostic significance of DUSP10 in LGG and found that the high-DUSP10 subset owned poorer prognosis when compared to the low-DUSP10 subset. The proportion of OS status of LGG patients was also investigated, and the results revealed that up-regulated DUSP10 expression was powerfully connected with inferior OS status. Receiver operating characteristics (ROC) curves and AUC values were implemented to validate the accurateness of DUSP10 in forecasting the OS of LGG patients. Besides, Cox regression analyses affirmed that DUSP10 was an independent prognostic factor of LGG. Analogical results were discovered in the CGGA and GSE61374 datasets.

GO-BP and KEGG analyses in the TCGA and CGGA datasets elucidated that DUSP10 expression was majorly associated with immune modulation, cell cycle, PI3K-Akt and JAK-STAT signaling pathways. Additionally, GSEA analysis explicated that the high-DUSP10 subset was mainly linked to immune responses and cancer-associated signaling pathways. Thus, DUSP10 might promote the malignant progression of LGG by activating the cancer-connected signaling pathways.

**Figure 8:**
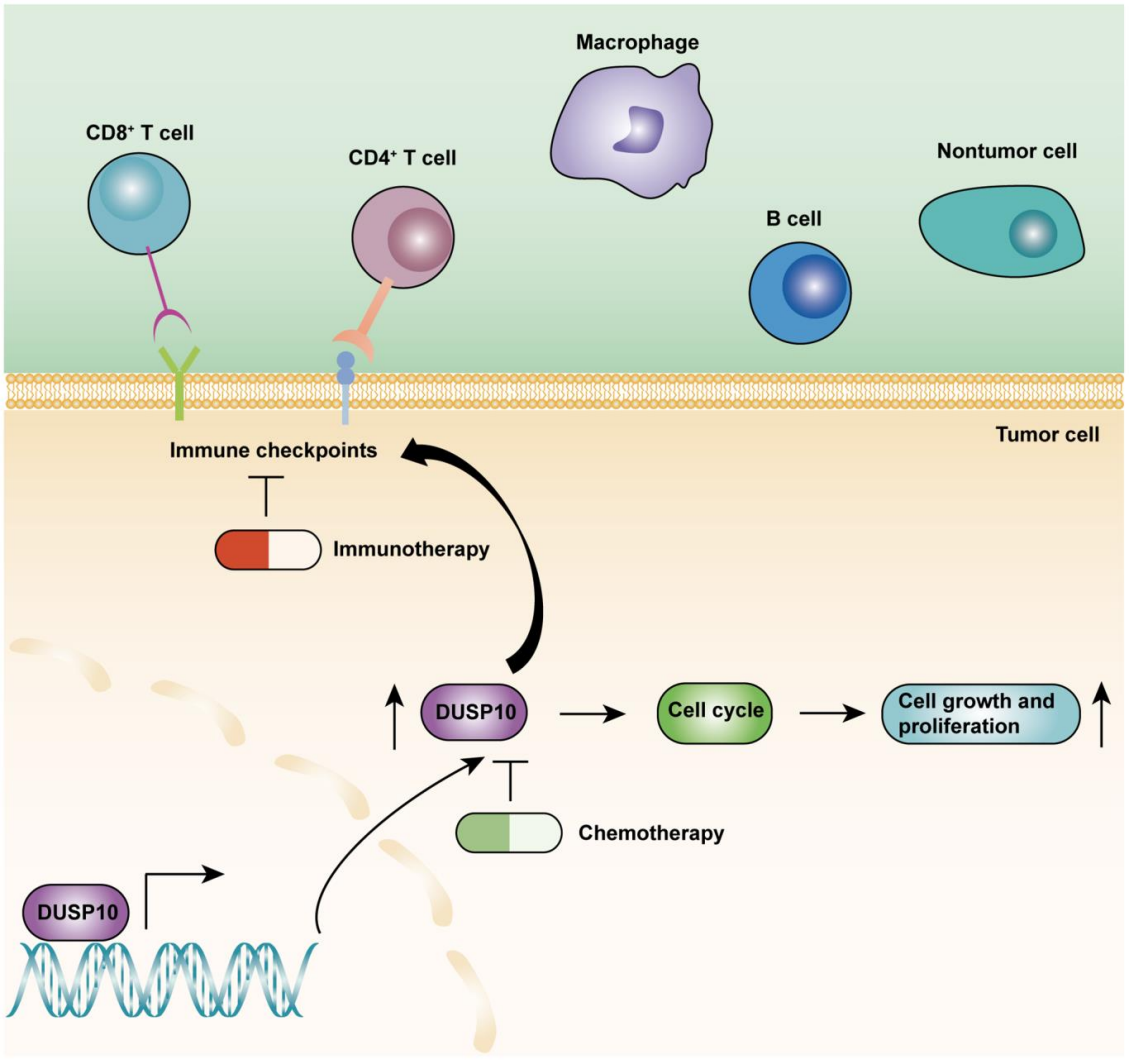
The underlying biological mechanisms of DUSP10 in LGG.

The TME mainly included tumor cells, tumor-related stromal cells, and immune cells [19]. Multiple studies confirmed that TME played a pivotal part in the malignant progression, prognosis, and immunotherapy response of LGG [20, 21]. Therefore, it is necessary to detect the connection between the DUSP10 expression and TME in LGG. The ssGSEA algorithm was employed to examine the difference in immune-interrelated signatures between the two subsets in the TCGA and CGGA cohorts. The ESTIMATE and CIBERSORT algorithms were also implemented to identify the composition of TME and TIICs in the two subtypes. These results declared that DUSP10 expression was forcefully associated with the immune infiltration in LGG. Moreover, the results of single-cell analysis disclosed that DUSP10 was closely interrelated with the LGG immune microenvironment. Recently, immunotherapy has become a novel treatment for tumors by activating specific immune cells in the tissue microenvironment [22–24]. Particularly, ICB have become a new immunotherapy drug to treat different types of tumors and provided good curative effects [25–27]. Thence, we assessed the connection between DUSP10 expression and ICPGs expression in LGG patients and detected that DUSP10 expression was positively related to the expression of some common ICPGs in the TCGA and CGGA datasets. Importantly, we detected that ICB therapy was more effective in high-DUSP10 subgroup than in low-DUSP10 subgroup by performing the TIDE algorithm. Additionally, the somatic mutation and CNA analyses suggested that the high-DUSP10 expression subset owned higher TBM and CNA burden than the low-DUSP10 expression subset. Thus, DUSP10 might play a vital value in the immunotherapy of LGG patients. The underlying roles of DUSP10 in LGG were displayed in Figure 8.

Currently, TMZ is the most commonly used in the treatment of glioma patients. However, its efficacy remains restrained. Thus, it is urgently needed to examine the new therapeutic drugs for LGG patients. The chemotherapeutics sensitivity analysis determined that the high-DUSP10 subset was more effective to chemotherapeutics, such as PIK-93, TGX221, AKT inhibitor VIII, MK-2206, bortezmib, and MG-13, than the low-DUSP10 subset. Hence, DUSP10 might represent a potential predictor for the chemosensitivity of patients with LGG.

By knocking down the DUSP10, we ascertained that the LGG cell proliferation ability was significantly reduced. Therefore, DUSP10 may be an effective target for LGG treatment in the near future. However, DUSP10 might be not an optimal drug target because of the formation of LGG involves activation of a variety of oncogenes and signaling pathways. The best therapeutic effect could be achieved only by systematically assessing the LGG patient's condition and developing individualized treatment plan. Additionally, some limitations existed in our research. Firstly, more independent LGG cohorts should be included to check the prognostic significance of DUSP10 in LGG. Secondly, the specific functions of DUSP10 in LGG should be detected by exploiting *in vivo* studies in the future.

## CONCLUSION

The study illustrated that DUSP10 was a powerful prognostic factor and strongly related to cellular proliferation in LGG. DUSP10 might become an effectual target/therapeutic agent for patients with LGG.

## METHODS

### Data gathering and collating

The DUSP10 expression, survival, clinical, and TMB data in 33 tumors were attained from the TCGA database. Additionally, the DUSP10 expression data of normal tissue was acquired from Genotype-Tissue Expression (GTEx).

Adjacently, three independent LGG cohorts, TCGA, CGGA (CGGA_325), and GSE61374 cohorts, were employed in this research. The mRNA expression, survival, and clinical data of LGG samples from the three independent cohorts were obtained from TCGA, CGGA, and Gene Expression Omnibus (GEO) databases. RNA-seq expression data from TCGA and CGGA cohorts in fragments per kilobase million format, was transformed into transcripts per kilobase million values and then transformed by log2. Similarly, the robust multichip averaging analysis (RMA)-processed values of GSE61374 were transformed by log2 to permit easier comparison. Additionally, the single-cell RNA-seq dataset GSE167960 was acquired from GEO website.

### Inclusion criteria for samples

LGG samples were included if they had: (1) WHO grade information; (2) gene expression information; (3) OS > 1 month. In total, 477 (Supplementary Table 1), 170 (Supplementary Table 2), and 137 (Supplementary Table 3) LGG samples were filtered out from TCGA, CGGA, and GEO datasets, respectively. Additionally, we included LGG samples with OS of < 1 month for pan-cancer analysis of DUSP10 to assure the consistency of survival information in 33 tumor types.

### Prognostic role of DUSP10

LGG samples were categorized into high-DUSP10 and low-DUSP10 subsets in line with median values of DUSP10 expression in the three cohorts. The KM analysis was exploited to determine the OS of LGG patients in high-DUSP10 and low-DUSP10 subsets. Additionally, the survival state ratio, ROC curves, and AUC values were employed to check the exactness of DUSP10 expression in forecasting the prognosis of LGG patients in the three datasets. Afterwards, we employed Cox regression analyses in the three datasets to examine whether DUSP10 expression was an independent biomarker of LGG patients.

### Functional enrichment and gene set enrichment analysis

Under the criteria of |log2FC| of > 0.5 and false-discovery rate (FDR) of < 0.05 [28, 29], we exploited R package limma to select the DEGs between the low-DUSP10 and high-DUSP10 subsets [30]. On the grounds of DEGs, we executed GO-BP and KEGG analyses by utilizing R package clusterProfiler [31]. GSEA analysis was implemented to detect the obviously enriched molecular pathways in the two subsets [32]. With the standards of normalized enrichment score (NES) >1, *p* < 0.05, and FDR < 0.25, we identified the significant enriched molecular pathways in the two subsets.

### Immunological characteristics and single-cell analysis

The immunological features including immune signatures, abundances of stromal and immune cells, and ICPGs expression level were evaluated. First, the ssGSEA algorithm was applied to ascertain the differential abundance of 29 immune-associated signatures, which were obtained from previous studies [33, 34], in low-DUSP10 and high-DUSP10 subsets. The ESTIMATE algorithm was conducted to investigate the abundance of immune cells, stromal cells, and tumor purity according to the expression profiles of LGG patients [35]. Four kinds of scores, including ESTIMATE score (representing nontumor composites), stromal score (representing the abundance of stromal cells), immune score (representing the abundance of immune cells), and tumor purity, were measured. Whereafter, the CIBERSORT algorithm was executed to quantify the infiltration level of TIICs in line with the gene expression data of LGG patients [36]. Additionally, we selected 25 ICPGs according to previous research [37], and investigated their correlation with DUSP10 expression. The GSE167960 dataset was exploited to inspect the correlation between DUSP10 and LGG immune microenvironment.

### Gene variation analysis

The RCircos tool was utilized to recognize and exhibit the conspicuous deletions and amplifications in the whole genome between low- and high- DUSP10 expression subgroups [38]. We performed the Maftools and GenVisR [39, 40] to account for and display variation types and frequencies of genes between low-DUSP10 and high-DUSP10 subgroups. The TMB, as a newly developing biomarker for forecasting the response to immunotherapy, reveals the total number of nonsynonymous mutations. The conjunction between DUSP10 expression and TMB level in 33 kinds of cancer was ascertained by performing R package fmsb. Afterwards, the association between the DUSP10 expression and TMB level was detected by conducting the R package ggplot2 in the independent LGG TCGA dataset.

### Evaluation of DUSP10 expression and treatment responses

The TIDE algorithm was implemented to detect the reaction of LGG patients to immunotherapy according to the expression data of LGG patients. Moreover, the sensitivity difference to several chemotherapeutic drugs, such as PI3K/AKT inhibitors (PIK-93, TGX221, AKT inhibitor VIII, and MK2206) and a proteasome inhibitor (bortezomib and MG-132), between high-DUSP10 and low-DUSP10 subtypes was explored by using R package pRRophetic [41].

### Cell culture and transfection

Three LGG lines, including SW1088, SW1783, and BT142, were obtained from the American Type Culture Collection. Normal human astrocyte (NHA) cell line was purchased from Culture Collection of the Chinese Academy of Sciences (Shanghai, China). SW1783 and SW1088 cell lines were incubated with Leibovitz’s L-15 medium and 10% fetal bovine serum (Gibco). BT142 and NHA cell lines were incubated with Dulbecco’s modified Eagle’s medium/F12 medium. All cell lines were incubated at 5% CO2 and 37°C. SW1088 cell lines were transfected with lentiviral vector containing DUSP10 shRNA (5′-CAATGAACCAAGCCGAGTGAT-3′) or negative control (NC) vector at a multiplicity of infection of 10. Polybrene was employed to elevate transfection efficiency and puromycin was applied to filter out positive cells.

### Western blot analysis

LGG and para-cancerous tissue samples (*n* = 6 each) were gathered from the Second Affiliated Hospital of Nanchang University. We extracted brain tissues and cell lysates by exploiting radioimmunoprecipitation assay buffer (Solarbio, China) mixed with proteinase inhibitors. Then, we separated lysates by utilizing 10% SDS-PAGE and transferred it to PVDF membranes. Then, the membranes were incubated with primary antibodies, including DUSP10 (1:1000, DF4694 affinity Biosciences, Proteintech, China) and glyceraldehyde-3-phosphate dehydrogenase (GAPDH) (1:20000, 60004-1-lg, Proteintech, China), and the relevant secondary antibodies. Eventually, the bands on the membranes were visualized by conducting a GV6000M imaging system (GelView 6000pro).

### Quantitative real-time PCR

Total RNA was isolated from cells with the Simply P Total RNA Extraction Kit (Bioflux, China) and reverse-transcribed it into complementary DNA with HiScript III-RT SuperMix (Vazyme, China). Subsequently, the values were managed with the 2^−ΔΔCT^ method. Primer sequences of genes were as follows: DUSP10 forward, 5′-ATACCAATGAACCAAGCCGAGT-3′; DUSP10 reverse, 5′-TCTTGGAGCTGGAGGGAGTTG-3′; GAPDH forward, 5′-GGTGTGAACCATGAGAAGTATGA-3′; and GAPDH reverse, 5′-GAGTCCTTCCACGATACCAAAG-3′.

### CCK-8 assay

Transfected SW1088 cells (2 × 10^3^ peer well) were plated in 96-well plates and cultured for 5 days. Cell proliferation was examined by Cell Counting Kit 8 assay (Glpbio, GK10001) according to the protocol.

### Colony formation assay

Transfected SW1088 cells were seeded in 6-well plates at 2 × 10^3^ peer well and incubated for 2 weeks. Subsequently, 0.1 % crystal violet stain solution was implemented to stain the cells, and the number of colonies was quantified by ImageJ.

### EdU assay

Transfected SW1088 cells (2 × 10^4^) were seeded in 24-well plates and incubate for 3 days. Subsequently, the cells were cultured with EdU reagent for 2 h and 4% paraformaldehyde and 0.5% Triton X-100 were employed to fix the cells. The Hoechst staining was exploited to stain the cells. The EdU incorporation rate was counted by ImageJ.

### Cell cycle analysis

Transfected SW1088 cells were fixed with 70% ethanol at 4°C overnight. Next, the cells were stained with RNase A containing propidium iodide (Suzhou, China). We ascertained the cell cycle distribution by conducting flow cytometry.

### Statistical analysis

The KM analysis was executed to differentiate the prognosis between high-DUSP10 and low-DUSP10 subsets by using a two-sided log-rank test. The veracity of DUSP10 expression in predicting prognosis was verified by AUC values and ROC curves. The independent prognostic significance of DUSP10 was checked by exploiting Cox regression analyses. Comparison of these immune-associated factors, including 29 immune-connected signatures, TIICs, 25 ICPGs, TMB, and CNA burden, between the two subtypes was measured by the Student’s *t* test. Additionally, Pearson’s or Spearman’s correlation test was employed to examine the association between distributed variables. The sensitivity difference to anticancer drugs between the two subtypes was estimated by conducting the Wilcoxon signed-rank test. We performed all statistical analyses in R programming, version 4.1.0, SPSS Statistics, and GraphPad Prism 8. *P* < 0.05 was deemed to be significant.

### Availability of data and materials

The data used in the study can be acquired in the TCGA (https://portal.gdc.cancer.gov/), CGGA (http://www.cgga.org.cn/), and GEO (https://www.ncbi.nlm.nih.gov/geo/) websites.

## Supplementary Materials

Supplementary Figures

Supplementary Tables
