# Supplementary material for: DUSP10 is a novel immune-related biomarker connected with survival and cellular proliferation in lower-grade glioma
Source: Aging (Albany NY). 2023 Jun 29;15(12):5673–97. doi: 10.18632/aging.204821 (PMC10333081; doi:10.18632/aging.204821)
Supplement: Supplementary Figures [file aging-15-204821-s001.pdf]

SUPPLEMENTARY FIGURES

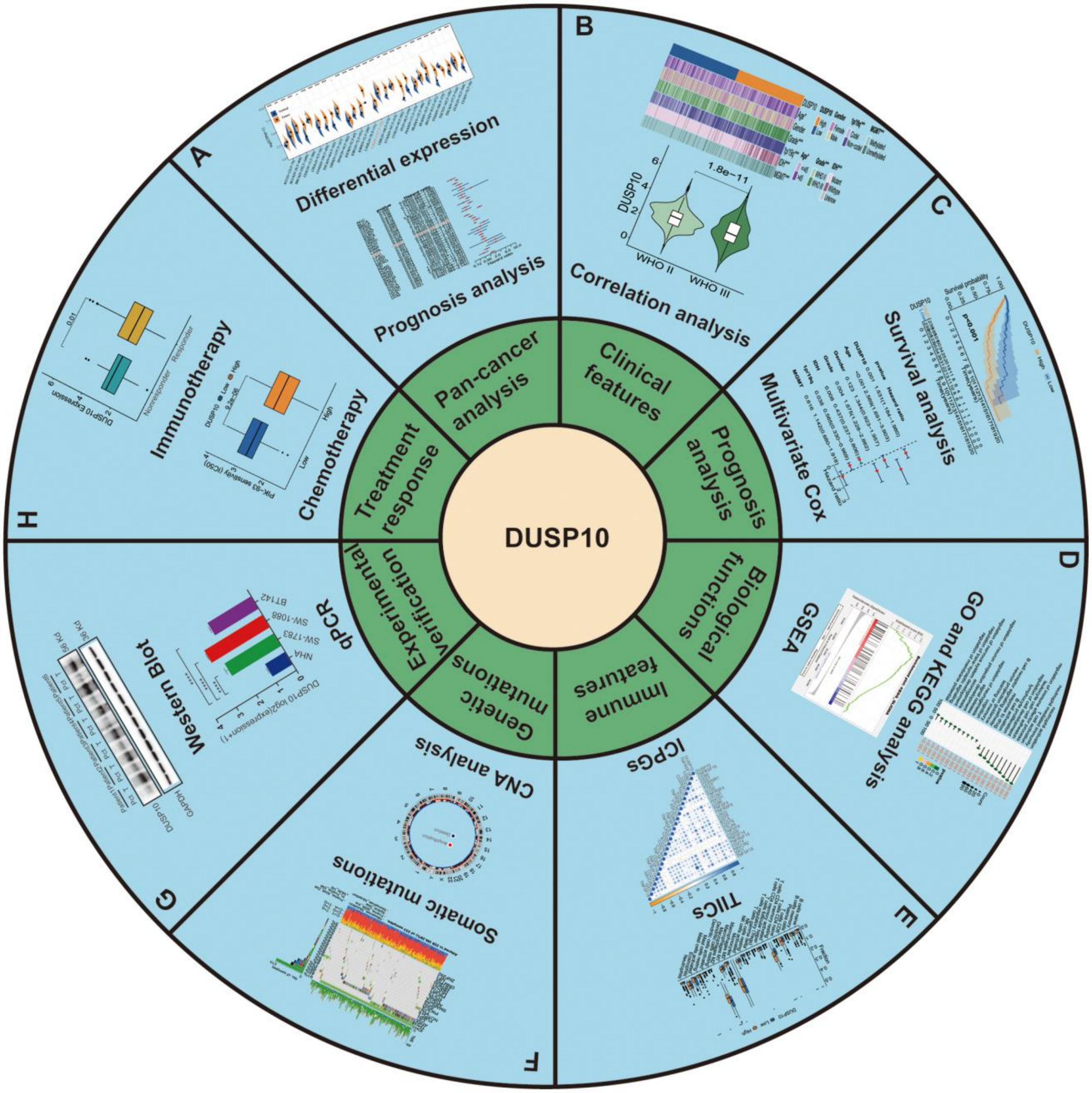

Supplementary Figure 1. Flow diagram of overall research. (**A**) Pan-cancer analysis. (**B**) Clinical features. (**C**) Prognosis analysis. (**D**) Biological functions. (**E**) Immune features. (**F**) Genetic mutations. (**G**) Experimental verification. (**H**) Treatment response of DUSP10 in LGG.

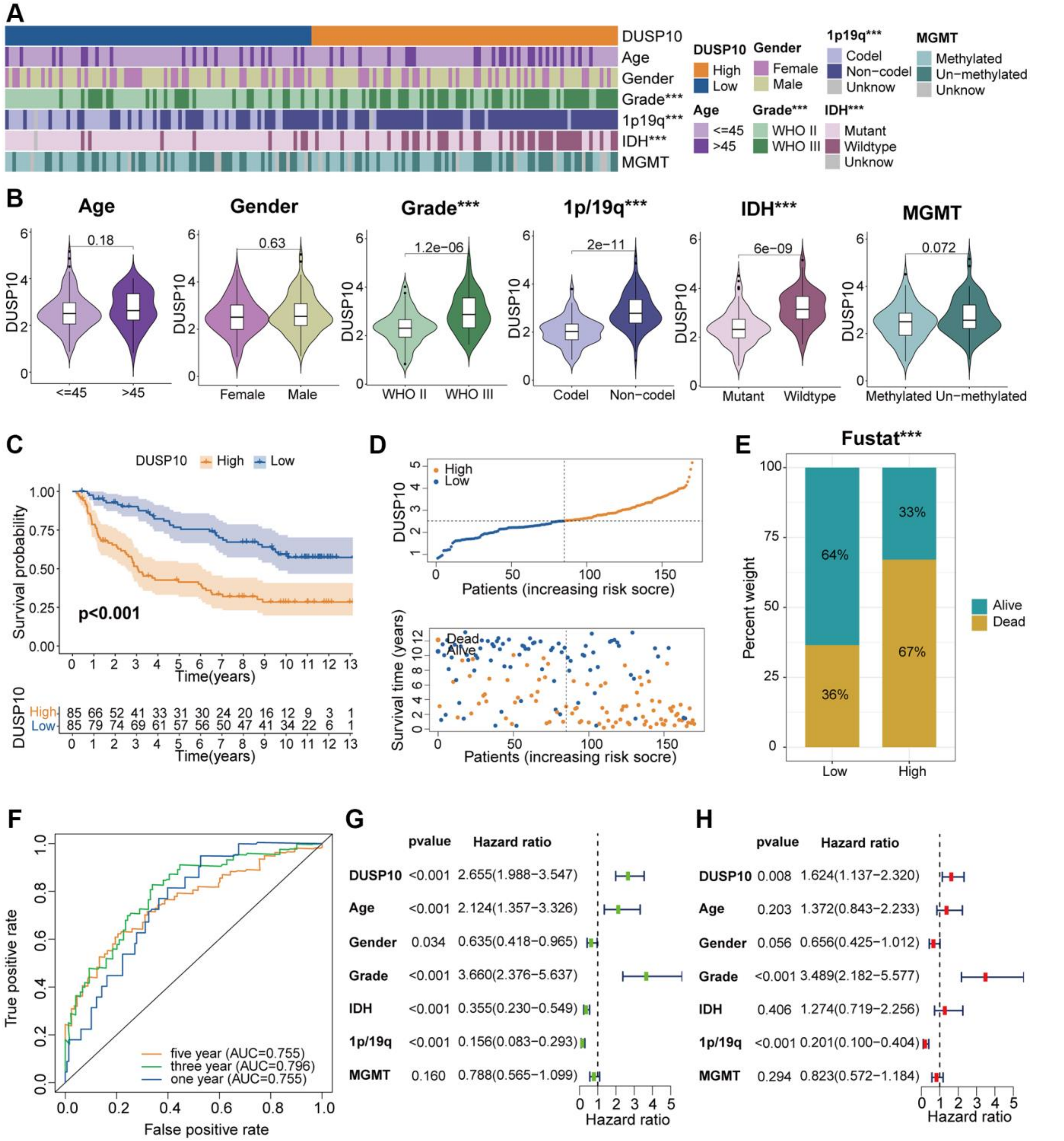

Supplementary Figure 2. Clinical correlation analysis of DUSP10 in CGGA. (**A**) Association between DUSP10 expression and clinical traits of LGG in CGGA. (**B**) Variance analysis of DUSP10 expression in different clinical features (including age, gender, grade, and 1p/19q, *IDH*, and MGMT statuses) in the CGGA dataset. (**C**) Prognostic analysis of high-DUSP10 and low-DUSP10 subtypes in the CGGA dataset. (**D**) Distribution of risk score, OS, and OS status of high-DUSP10 and low-DUSP10 subtypes in the CGGA dataset. (**E**) Different proportions of the living situation between the two subtypes. (**F**) ROC curves representing the predictive role of the risk score in CGGA. (**G**, **H**) Univariate and multivariate Cox analyses of DUSP10 expression and clinicopathological characteristics in CGGA. $^{*}P < 0.05$, $^{**}P < 0.01$, $^{***}P < 0.001$.

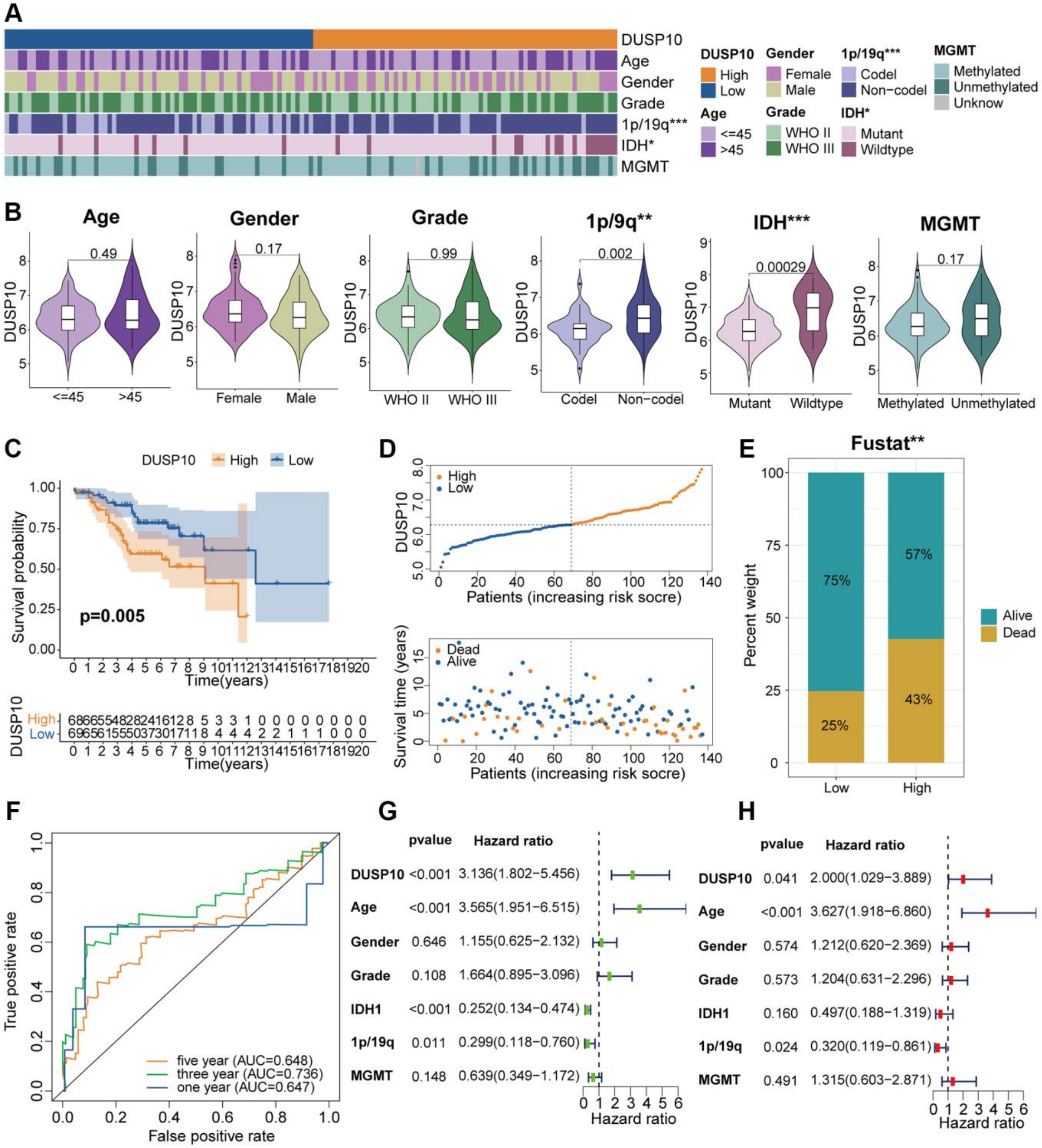

Supplementary Figure 3. Clinical correlation analysis of DUSP10 the GSE61374 dataset. (**A**) Association between DUSP10 expression and clinical traits of LGG the GSE61374 dataset. (**B**) Variance analysis of DUSP10 expression in different clinical features (including age, gender, grade, and 1p/19q, *IDH*, and MGMT statuses) in the GSE61374 dataset. (**C**) Prognostic analysis of high-DUSP10 and low-DUSP10 subtypes in the GSE61374 dataset. (**D**) Distribution of risk score, OS, and OS status of high-DUSP10 and low-DUSP10 subtypes in the GSE61374 dataset. (**E**) Different proportions of the living situation between the two subtypes. (**F**) ROC curves representing the predictive role of the risk score in GSE61374. (**G**, **H**) Univariate and multivariate Cox analyses of DUSP10 expression and clinicopathological characteristics in GSE61374. $^{*}P < 0.05$, $^{**}P < 0.01$, $^{***}P < 0.001$.

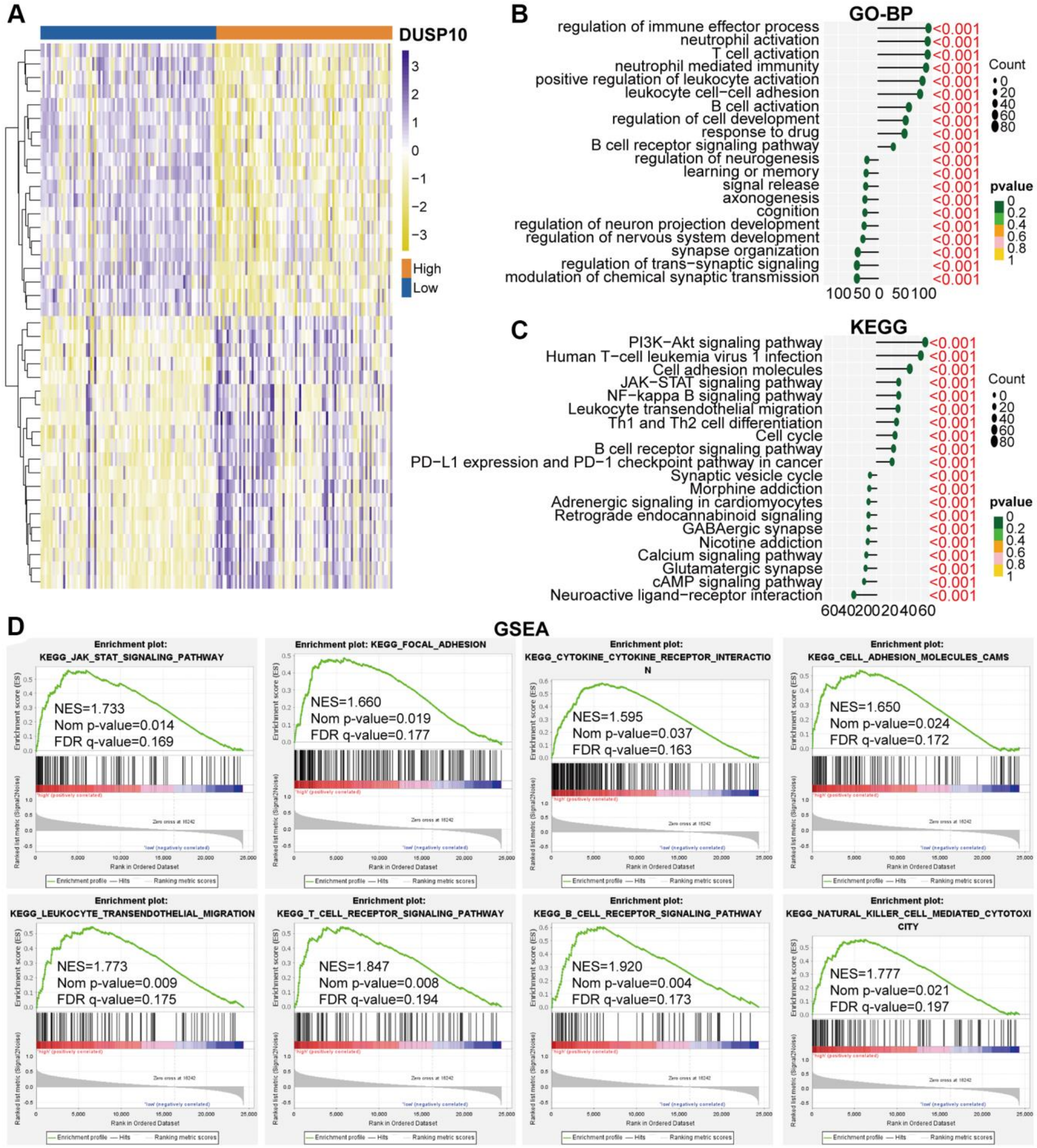

Supplementary Figure 4. Biological functions of DUSP10 in LGG in CGGA. (**A**) DEGs between the low-DUSP10 and high-DUSP10 expression LGG subgroups. (**B**, **C**) The GO-BP (**B**) and KEGG (**C**) analyses for DUSP10 in LGG patients in the CGGA dataset. (**D**) GSEA in the CGGA dataset.

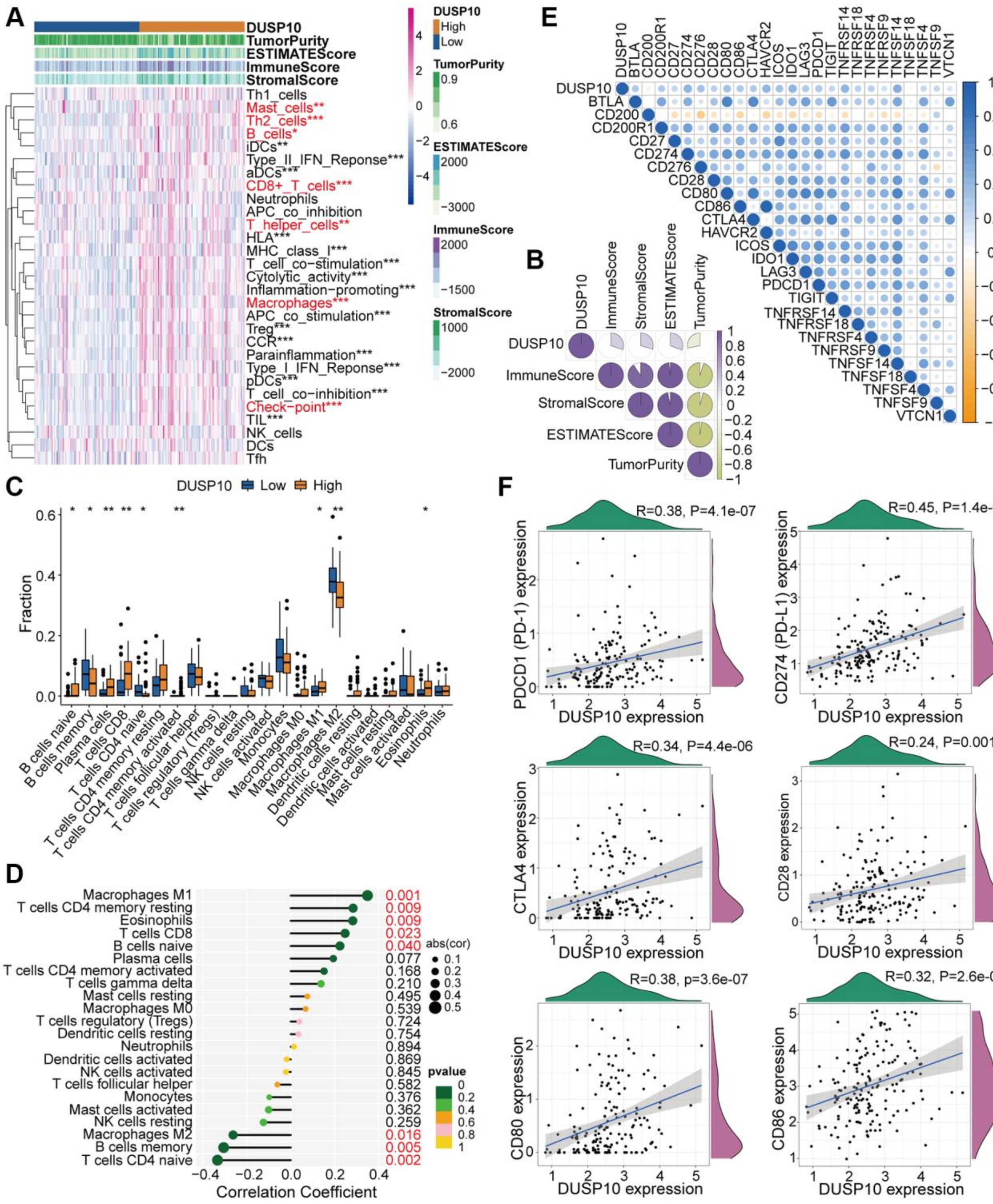

Supplementary Figure 5. Different TME and immunological characteristics of the low- DUSP10 and high-DUSP10 subtypes in CGGA. (**A**, **B**) Association between DUSP10 expression and 29 immune-associated signatures, ESTIMATE, immune, stromal scores, and tumor purity. (**C**) Comparisons of infiltration of 22 types of immune cells in the two subgroups. (**D**) Lollipop plots exhibited the relationship between DUSP10 expression and TIICs. (**E**, **F**) Co-expression analysis of DUSP10 and 25 ICPGs. $^{*}P < 0.05$, $^{**}P < 0.01$, $^{***}P < 0.001$.

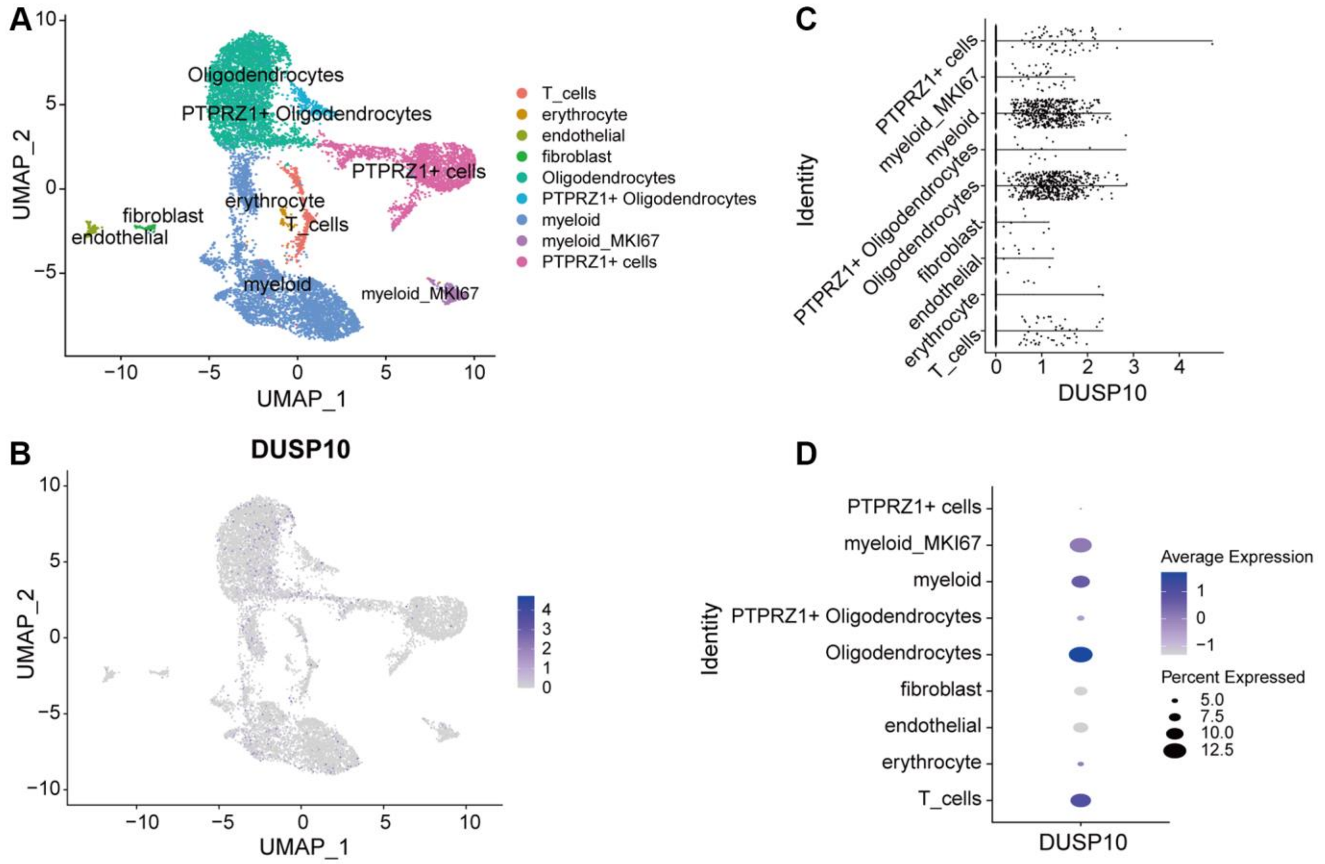

Supplementary Figure 6. Single-cell analysis of DUSP10 in GSE167960. (**A**, **B**) The cell types in LGG immune microenvironment and the expression distribution of DUSP10. (**C**, **D**) Expression levels of DUSP10 in immune cells.
