# Supplementary material for: DUSP10 is a novel immune-related biomarker connected with survival and cellular proliferation in lower-grade glioma
Source: Aging (Albany NY). 2023 Jun 29;15(12):5673–97. doi: 10.18632/aging.204821 (PMC10333081; doi:10.18632/aging.204821)
Supplement: Supplementary Tables [file aging-15-204821-s002.pdf]

SUPPLEMENTARY TABLES

Supplementary Table 1. Clinical features of LGG patients from TCGA.

Clinical features		Total (477)	%
Age	Age ≤ 45	287	60.17%
	Age >45	190	39.83 %
Gender	Female	216	45.28%
	Male	261	54.72%
Grade	WHO II	231	48.43%
	WHO III	246	51.57%
1p/19q	Non-codel	321	67.30%
	Codel	156	32.70%
IDH	Mutant	389	81.55%
	Wildtype	85	17.82%
	Unknow	3	0.63%
MGMT	Unmethylated	82	17.19%
	Methylated	395	82.81%

Supplementary Table 2. Clinical features of LGG patients from CGGA.

Clinical features		Total (170)	%
Age	Age ≤ 45	129	75.88%
	Age >45	41	24.12%
Gender	Female	65	38.24%
	Male	105	61.76%
Grade	WHO II	97	57.06%
	WHO III	73	42.94%
1p/19q	Non-codel	113	66.47%
	Codel	55	32.35%
	Unknow	2	1.18%
IDH	Mutant	125	73.53%
	Wildtype	44	25.88%
	Unknow	1	0.59%
MGMT	Unmethylated	70	41.18%
	Methylated	84	49.41%
	Unknow	16	9.41%

Supplementary Table 3. Clinical features of LGG patients from GSE61374.

Clinical features		Total (137)	%
Age	Age ≤ 45	83	60.58%
	Age >45	54	39.42%
Gender	Female	53	38.69%
	Male	84	61.31%
Grade	WHO II	61	44.53%
	WHO III	76	55.47%
1p/19q	Non-codel	100	72.99%
	Codel	37	27.01%
IDH	Mutant	115	83.94%
	Wildtype	22	16.06%
MGMT	Unmethylated	38	27.74%
	Methylated	98	71.53%
	Unknow	1	0.73%
